# Oxidation Reaction Characteristics and Thermodynamic Analysis of Carbon Monoxide Following Gas Explosions

**DOI:** 10.3390/molecules31101729

**Published:** 2026-05-19

**Authors:** Shuai Wang, Gang Wang, Yashengnan Sun, Qiang Yuan, Jie Chen, Qian Jiang, Yanyan Zhu

**Affiliations:** 1 State Key Laboratory of Coal Mine Disaster Dynamics and Control, Chongqing University, Chongqing 400044, China; wangshuai_xust@163.com (S.W.); jiechen023@cqu.edu.cn (J.C.); 2 State Key Laboratory of Coal Mine Disaster Prevention and Control, Fushun 113122, China; 3 China Coal Technology and Engineering Group Shenyang Research Institute, Fushun 113122, China; 4 School of Safety Science and Engineering, Liaoning Technical University, Huludao 125000, China; sunnn360@163.com (Y.S.); mrjiang129@163.com (Q.J.); 13591991745@163.com (Y.Z.)

**Keywords:** gas explosion, CO elimination, hopcalite catalyst, thermodynamic analysis, equilibrium constant

## Abstract

The high concentration of CO generated in confined spaces following a gas explosion constitutes the primary lethal factor, and its rapid elimination represents a critical technical bottleneck in emergency rescue operations. This study systematically investigates the confined thermodynamic characteristics of CO catalytic oxidation over hopcalite across a wide temperature range of 15–65 °C. Based on the ideal gas assumption and constant-volume boundary conditions, the thermodynamic processes were classified into two categories: constant-volume variable-temperature and constant-temperature constant-volume. The influence of temperature on enthalpy change, heat release, entropy change, and the chemical equilibrium constant was quantitatively examined. The results demonstrate that the total enthalpy change and heat release remained negative throughout the entire temperature range, exhibiting a trend of “initial increase, subsequent decrease, followed by a slight rise”, with the maximum exothermic value observed at 25 °C. The total entropy change was persistently negative across the full temperature range; the positive offset contribution of the physical entropy change induced by temperature elevation was negligible, resulting in a consistently high absolute value of the total entropy change. The logarithm of the standard equilibrium constant decreased linearly with increasing temperature yet remained as high as 180.48 at 65 °C, indicating that the reaction maintains an extremely strong thermodynamic spontaneity and a nearly complete conversion limit under all tested conditions.

## 1. Introduction

Gas explosions represent one of the most severe types of major accidents in the field of coal mine safety production in China. Following an explosion, high concentrations of carbon monoxide (CO) at the percentage level are rapidly generated within the confined underground spaces [1,2,3]. Carbon monoxide is highly toxic and exhibits prolonged retention, making it the primary cause of post-explosion underground casualties. Moreover, its flammable and explosive nature introduces substantial secondary safety risks during emergency rescue operations [4,5]. The rapid, efficient, and stable elimination of high-concentration CO within confined and enclosed spaces after a gas explosion constitutes a core technical bottleneck for reducing accident-related fatalities and ensuring the safety of emergency response. Consequently, investigating the relevant reaction mechanisms and thermodynamic characteristics holds significant engineering practical value and immediate relevance.

Among the various CO elimination technologies, catalytic oxidation has emerged as the preferred technical pathway due to its mild reaction conditions, high conversion efficiency, and absence of secondary pollution [6,7,8]. Xie et al. [9] synthesized highly active Co_3_O_4_ nanorods by calcining a CoHCO_3_ precursor; the catalyst achieved complete CO conversion at 77 °C and maintained excellent activity even under humid atmospheres, a performance attributed to the abundance of highly active Co^3+^ species on the surface. Xie et al. [10] prepared CuO/CeO_2_ catalysts with different morphologies using a combined hydrothermal and impregnation method, among which spindle-shaped catalysts exposing specific crystal facets exhibited both a lower CO conversion temperature and a broader operational temperature window. Mahalik et al. [11] fabricated Cu–Cu_2_O composite catalysts via a redox method, which demonstrated superior performance in CO oxidation compared to pure Cu_2_O. Liu et al. [12] hydrothermally synthesized α-Fe_2_O_3_ catalysts with various morphologies and found that nanorod-like samples displayed the highest catalytic activity due to the high iron atom density on the exposed (110) facet. The CuMn-based hopcalite catalyst, characterized by its excellent ambient-temperature catalytic activity, low cost, and ease of preparation, exhibits considerable application potential [13,14,15]. To date, domestic and international research on hopcalite-catalyzed CO oxidation has predominantly focused on catalyst modification and optimization [16,17,18,19], catalytic reaction kinetics [13,20,21], and the influence of operating parameters such as humidity and space velocity on catalytic activity [21,22,23]. Dey et al. [24] prepared CuMnO_x_ catalysts via a co-precipitation method using KMnO_4_, Na_2_CO_3_, and KOH as precipitants; the results indicated that the catalyst obtained with KMnO_4_ exhibited the highest activity, achieving complete CO oxidation at low temperatures. Xin et al. [25] developed Cu–Mn–Sn composite oxide catalysts tailored for coal mine environments and investigated the effects of CO concentration (1–7%), temperature (25–300 °C), and water poisoning (0–100%). The results demonstrated that the CO concentration could be reduced to below 0.55% within 248 s, with an elimination efficiency reaching 99.99% at 300 °C. Biemelt et al. [26] prepared a water-resistant hopcalite catalyst via flame spray pyrolysis (FSP) by dispersing a mixture of oleates, 2-ethylhexanoates, and nitrates in a surfactant. Wang et al. [27] employed catalytic oxidation for the low-temperature conversion of CO to CO_2_ and synthesized Cu–Mn catalysts via co-precipitation, examining the effects of the Cu/Mn ratio and reaction temperature on catalytic efficiency. Guo et al. [28] prepared a CuMn–HZSM-5 composite catalyst using an impregnation method; the optimized sample achieved a NO conversion rate of 90% and a CO conversion rate of 100% at 175 °C. These studies have confirmed the excellent CO conversion capability of this catalyst under ambient temperature and pressure conditions, thereby laying a foundation for engineering applications in underground scenarios.

However, existing research has largely focused on catalytic reaction performance and kinetic mechanisms within open-flow systems. A significant theoretical gap remains regarding thermodynamic investigations specific to the rigid, confined, constant-volume scenarios that are characteristic of post-gas-explosion environments. On the one hand, few studies have systematically elucidated the evolution of key thermodynamic parameters, such as enthalpy change, entropy change, and Gibbs free energy change, for the CO catalytic oxidation reaction across the wide temperature range of 15–65 °C commonly encountered in underground mines. On the other hand, a systematic theoretical analysis is still lacking with respect to the delineation of thermodynamic processes under constant-volume conditions, the thermodynamic characteristics of distinct reaction stages, and the regulatory mechanism of temperature on the reaction equilibrium limit for strongly exothermic reactions. This deficiency hinders the provision of precise thermodynamic theoretical support for the engineering application of CO catalytic elimination in confined spaces.

Based on the above considerations, this study employed hopcalite as the catalyst for CO oxidation. Targeting the uniquely rigid constant-volume confined enclosed space without mass exchange subsequent to mine gas explosions, a constant-volume CO catalytic oxidation experimental system was established, and systematic catalytic experiments were conducted across the temperature range of 15–65 °C. Different from the thermodynamic research of existing open-flow systems, this study adopted the ideal gas assumption and constant-volume reaction boundary conditions suitable for confined post-gas-explosion environments. Two thermodynamic processes, namely constant-volume variable-temperature and constant-temperature constant-volume, are classified to revise the calculation methods for total enthalpy change and total entropy change. The regulatory law of temperature on reaction thermodynamic parameters is quantitatively clarified, and the non-monotonic evolution trend of thermodynamic performance characterized by low-temperature promotion and high-temperature attenuation is revealed in the constant-volume confined system. The research results can enrich the thermodynamic theoretical system of CO catalytic elimination in confined spaces, and provide theoretical support and engineering data for the efficient disposal of CO in underground coal mine emergency rescue.

## 2. Results and Discussion

### 2.1. Experimental Characterization

**Figure 1:**
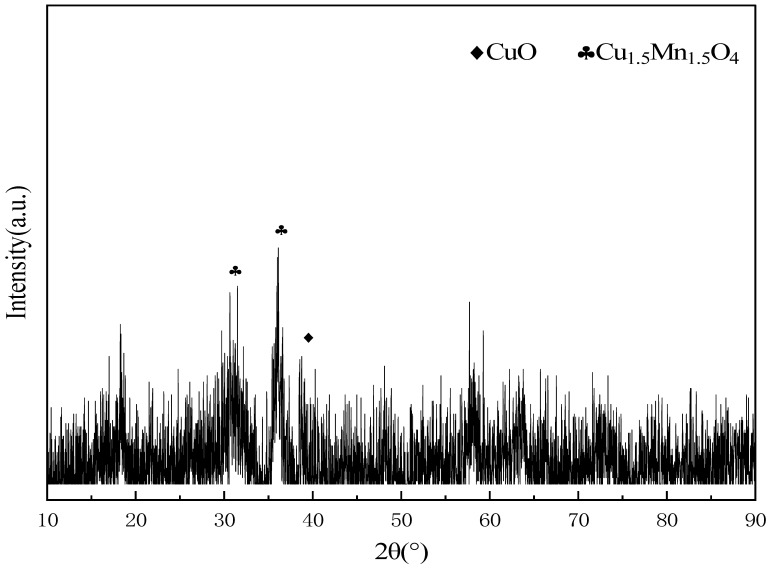
XRD pattern of the hopcalite catalyst.

The XRD pattern of the hopcalite catalyst is presented in Figure 1. Low-intensity characteristic diffraction peaks of the Cu_1.5_Mn_1.5_O_4_ spinel phase and CuO phase can be observed, while no obvious peaks corresponding to MnO_x_ phases were detected, which conforms to the typical structural features of hopcalite catalysts. It is speculated that a large amount of MnO_x_ exists in the catalyst in an amorphous form. The amorphous structure can supply abundant surface defects and active sites, and exert a synergistic effect with crystalline Cu_1.5_Mn_1.5_O_4_ and CuO phases. Together, they constitute the core active structure for CO catalytic oxidation, which provides a stable and reliable material foundation for the subsequent thermodynamic investigation of CO catalytic oxidation in confined systems.

**Figure 2:**
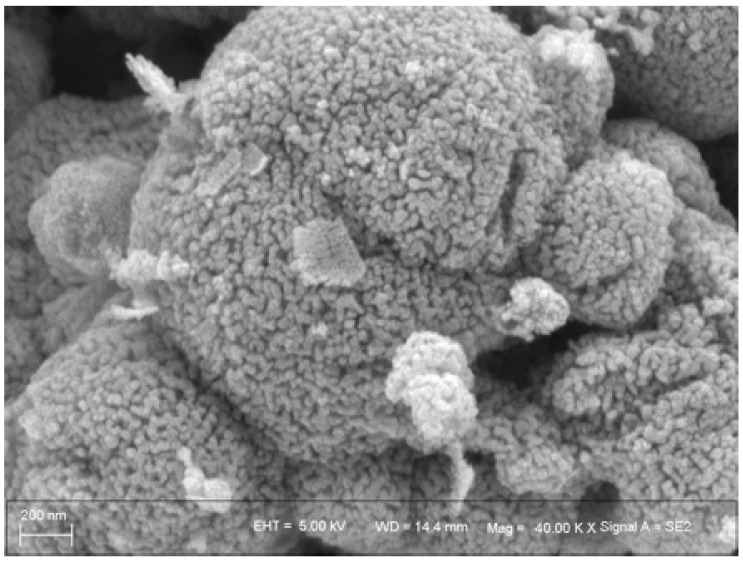
SEM image of the hopcalite catalyst.

The SEM image of the catalyst is shown in Figure 2, with a scale bar of 200 nm. It can be seen from the image that the hopcalite catalyst exhibited a uniform microspherical aggregate morphology. These aggregates, with a particle size of approximately 400–500 nm, were assembled from primary fine nanoparticles of about 20–30 nm. The surface of the microspheres was rough and porous, with no obvious large-scale agglomeration. This hierarchical structure not only ensures the full exposure of active sites, but also provides favorable channels for the adsorption and diffusion of CO and O_2_ molecules.

**Figure 3:**
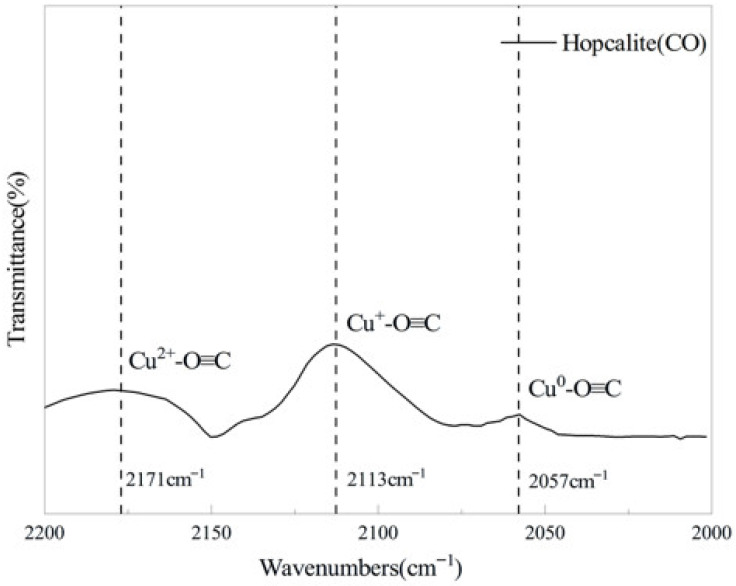
FTIR spectra of the hopcalite catalyst after CO adsorption.

The in situ spectroscopic scanning results of the catalyst after CO adsorption are displayed in Figure 3. A strong characteristic absorption band appeared in the range of 2100–2150 cm^−1^, which was assigned to the chemisorption peak of CO on the Cu^+^ active sites. The weak shoulder peaks at 2171 cm^−1^ and 2057 cm^−1^ corresponded to CO adsorption on the Cu^2+^ and Cu^0^ sites, respectively. The extremely low peak intensities indicate that Cu^+^ acts as the dominant core site for CO adsorption and activation on the catalyst surface.

Combined with the synergistic structure of the crystalline Cu_1.5_Mn_1.5_O_4_ spinel phase and amorphous MnO_x_ verified by XRD, the catalyst follows the Mars–van Krevelen (MvK) catalytic mechanism. CO is preferentially adsorbed and activated at Cu^+^ active sites. The activated CO reacts with the surface lattice oxygen of the catalyst to generate CO_2_ followed by desorption, meanwhile creating surface oxygen vacancies. Gaseous O_2_ is adsorbed and activated at oxygen vacancies to replenish lattice oxygen, thus completing the catalytic cycle. The efficient adsorption and activation process dominated by Cu^+^ sites directly determines the reaction rate and reaction progress, and further regulates macroscopic thermodynamic parameters such as system enthalpy change and heat release.

### 2.2. Thermodynamic Process Analysis

The experiments were conducted in a closed system at atmospheric pressure over a temperature range of 15–65 °C, with the maximum system pressure not exceeding 0.12 MPa. Neither extremely low temperatures nor high-pressure conditions were encountered. Under these circumstances, the intermolecular forces among the components of the gas mixture—CO, O_2_, CO_2_, and N_2_—are negligible, and the pressure–volume–temperature (PVT) behavior of the mixture closely approximates that of an ideal gas. According to the applicability criterion for ideal gases in engineering thermodynamics, the compressibility factor Z of small-molecule gases including CO, O_2_, CO_2_, and N_2_, and all approach 1 at normal temperature and low pressure (*p* ≤ 0.5 MPa). The compressibility factor Z of the experimental system, calculated via partial pressure weighting of the mixed gas, ranges from 0.998 to 1.002. Its relative deviation from the ideal gas condition (Z = 1) is less than 0.2%, which is far lower than the allowable error range in engineering thermodynamic calculations (<1%). Thus, the effect of system non-ideality on the calculation of thermodynamic parameters can be neglected. Therefore, the gas mixture was treated thermodynamically as an ideal gas throughout this study, an approach that conforms to the applicable boundary conditions of the ideal gas assumption and the standard practices of engineering thermodynamic calculations.

**Figure 4:**
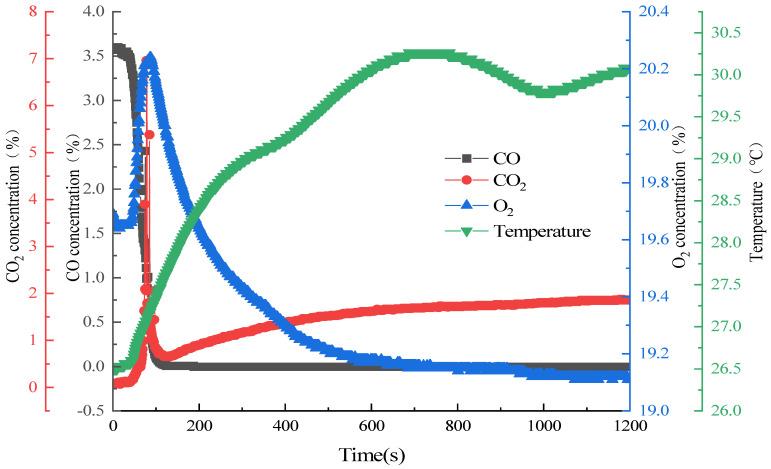
Temporal profiles of gas concentrations and system temperature. Conditions: CO concentration = 3%, T = 25 °C.

Nitrogen (N_2_) served as an inert balance gas; it did not participate in the chemical reaction and acted solely as a diluent within the system. All experiments were carried out in a rigid, sealed elimination vessel with a fixed internal volume. Consequently, constant-volume conditions were adopted as the fundamental boundary constraint for all experimental runs. Taking the experiment conducted at an initial temperature of 25 °C as a representative example, the temporal evolution of gas concentration and system temperature during the reaction process is illustrated in Figure 4.

As shown in Figure 4, the evolution of gas concentrations and system temperature during the reaction proceeds through distinct stages. The period from 0 to 45 s corresponds to an induction phase, during which the CO and CO_2_ concentrations remain essentially stable, whereas the O_2_ concentration declines rapidly. No significant catalytic oxidation reaction occurs in this phase; the consumption of O_2_ is primarily attributed to surface adsorption on the catalyst and the replenishment of oxygen vacancies. The interval from 45 to 100 s represents the reaction initiation stage, characterized by a rapid decrease in CO concentration and a pronounced increase in CO_2_ concentration, signifying the onset of the primary reaction. A transient recovery in O_2_ concentration was observed during this period, which can be ascribed to the increased relative proportion of O_2_ resulting from the rapid consumption of CO, coupled with the partial desorption of surface-adsorbed O_2_. Concurrently, the system temperature rises sharply. Beyond 100 s, the reaction enters a high-efficiency conversion stage: the O_2_ concentration declines continuously, the CO concentration decreases to an extremely low level and subsequently stabilizes, while the CO_2_ concentration exhibits a steady increase. In the later phase, after approximately 700 s, the O_2_ concentration decline becomes more gradual, and the system temperature initially stabilizes, then decreases, and finally rises slightly before the system ultimately attains a dynamic equilibrium.

Based on the real-time monitoring data from temperature sensors across all experimental runs, and considering the characteristic temperature variation observed in the representative 25 °C case, the six experimental conditions were classified into two distinct thermodynamic processes. For initial system temperatures of 15, 25, 35, 45, and 55 °C, the temperature difference between the system and the ambient environment is relatively small, resulting in a low natural heat dissipation rate. Under these conditions, the rate of heat release from CO oxidation consistently exceeds the rate of heat dissipation, leading to a pronounced variation in system temperature as the reaction progresses. Accordingly, these reactions are designated as constant-volume variable-temperature processes. In contrast, for the 65 °C case, the initial temperature difference relative to the ambient environment is significantly larger, yielding a higher natural heat dissipation rate. Under this condition, the rates of heat release and heat dissipation approach a dynamic balance, and the system temperature reading remains essentially constant. Consequently, this reaction is designated as a constant-temperature constant-volume process. All subsequent calculations and analyses of thermodynamic parameters presented in this work are predicated upon this process classification.

The core reaction investigated in this study was the hopcalite-catalyzed oxidation of carbon monoxide, as expressed in Equation (1):

2CO (g) + O_2_ (g) → 2CO_2_ (g) (1)

The temperature of 25 °C (298.15 K) corresponds to the thermodynamic standard state and also constitutes one of the core experimental conditions investigated in this study. The fundamental thermodynamic parameters for each species involved in Reaction (1) at the standard state of 298.15 K and 100 kPa are listed in Table 1.

Based on the additivity principle of thermodynamic state functions, the molar thermodynamic quantities for the reaction at 25 °C were calculated as follows:

Standard molar enthalpy of reaction:

Δ_r_H_m_^θ^ (298 K) = 2 × (−393.509) − 2 × (−110.525) = −565.968 kJ·mol^−1^.

Standard molar reaction entropy:

Δ_r_S_m_^θ^ (298 K) = 2 × 213.74 − (2 × 197.674 + 205.138) = −173.006 J·mol^−1^·K^−1^

Standard molar Gibbs free energy change:

Δ_r_G_m_^θ^ (298 K) = −514.38 kJ·mol^−1^

Molar heat capacity difference at constant pressure:

Δ_r_C_p,m_^θ^ (298 K) = 2 × 37.11 − (2 × 29.144 + 29.355) = −13.42 J·mol^−1^ K^−1^

Standard equilibrium constant:

K_θ_ (298) = exp(Δ_r_G^m^ (298)/−R × 298.15) = exp (207.6)

These benchmark parameters indicate that the catalytic CO oxidation reaction at 298.15 K is strongly exothermic (Δ_r_H_m_^θ^ < 0) and is accompanied by a decrease in the degree of disorder of the system (Δ_r_S_m_^θ^ < 0). The Gibbs free energy change was significantly negative, implying an extremely strong thermodynamic spontaneity for the forward reaction. The standard equilibrium constant was exceedingly large in magnitude, demonstrating that the forward reaction can proceed to an exceptionally high extent, thereby enabling the nearly complete conversion of CO from a thermodynamic perspective. In the following sections, the influence of temperature on the aforementioned thermodynamic parameters across the range of 15–65 °C will be systematically analyzed based on these benchmark data.

**Figure 5:**
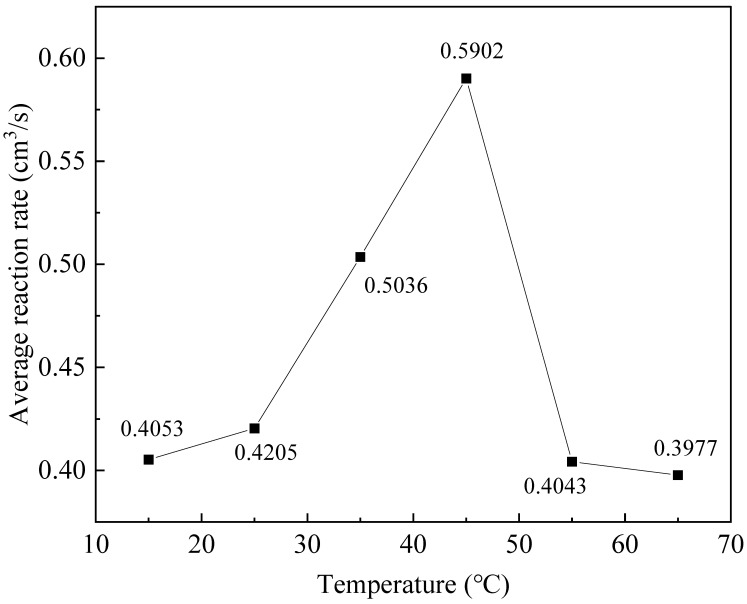
Average reaction rate of CO catalytic oxidation at different temperatures (lines are guides for the eye).

To further understand the kinetic characteristics corresponding to the above thermodynamic processes, the average CO reaction rates at different temperatures were calculated and analyzed, as depicted in Figure 5. The average rate exhibited a volcanic variation trend with increasing temperature, which increased gradually and reached the maximum at 45 °C, indicating that the catalytic activity is significantly enhanced with rising temperature. However, the rate decreased obviously at 55 and 65 °C, which is attributed to the weakened adsorption capacity of active sites and the constraint of fixed reactant dosage in the constant-volume system.

Notably, the reaction rate peaked at 45 °C, while the thermodynamic heat release and enthalpy change were demonstrated to peak at 25 °C in the subsequent analysis. This discrepancy confirms the dual regulatory effect of temperature in the confined constant-volume system: the kinetic activity is positively promoted by temperature, whereas the thermodynamic reaction extent is restricted by the fixed initial reactant amount. The coupling between kinetic characteristics and thermodynamic processes dominates the overall reaction behavior of CO catalytic oxidation in this study.

### 2.3. Influence on Enthalpy Change (*Δ*H) and Heat Release

Enthalpy is a fundamental state function that characterizes the thermal effect of a system. The molar reaction enthalpy change of the CO catalytic oxidation reaction governs the theoretical thermal effect of the process, whereas the actual amount of heat released by the system serves as a key engineering parameter for evaluating the heat release characteristics during CO elimination. All experiments in this study were conducted within a rigid, sealed elimination vessel. In accordance with the thermodynamic process classification established in Section 2.2, the experimental runs at 15, 25, 35, 45, and 55 °C were designated as constant-volume variable-temperature processes, while the run at 65 °C was designated as a constant-temperature constant-volume process. Since this study focused on the overall thermodynamic behavior of a confined constant-volume system, total values were adopted rather than molar quantities.

#### 2.3.1. Calculation Method

The gas mixture is treated as an ideal gas, and its molar heat capacity at constant pressure, *C*_p,mix_, and molar heat capacity at constant volume, *C*_v,mix,_ are calculated as the weighted average of the volumetric fractions of the individual components, as expressed in Equation (2). The extent of reaction, *ξ*, is determined from the experimentally measured CO concentration values:

(2) $$\xi =\frac{n_{\mathrm{co},1}-n_{\mathrm{co},2}}{2}$$

where n_co,1_ and n_co,2_ denote the amount of substance (in mol) of CO at the initial state and at equilibrium, respectively.

(1) Constant-Volume Variable-Temperature Process

The average temperature of the initial and final states, T = (T_1_ + T_2_)/2, was adopted as the characteristic temperature, where T_1_ and T_2_ represent the thermodynamic temperatures (in K) at the onset of the reaction and at equilibrium, respectively. The molar reaction enthalpy change is corrected according to Kirchhoff’s law, as expressed in Equation (3):

(3) $${∆}_{r}H_{m}\left(T_{avg}\right)={∆}_{r}H_{m}\left(298.15K\right)+{∆}_{r}C_{p,m}\times \left(T_{avg}-298.15\right)$$

The total enthalpy change of the system consists of the chemical reaction enthalpy change and the physical enthalpy change, as expressed in Equation (4):

(4) $$\Delta H=\xi \times {∆}_{r}H_{m}\left(T_{avg}\right)+n_{mix}\times C_{p,mix}\times \left(T_{2}-T_{1}\right)$$

where ΔH denotes the total enthalpy change, J; ξ is the extent of reaction, mol; Δ*_r_H_m_* represents the molar reaction enthalpy, J·mol^−1^; n*_mix_* is the initial total amount of substance of the gas mixture, mol; and *C_p,mix_* is the constant-pressure heat capacity of the gas mixture, J·mol^−1^·K^−1^.

Under constant-volume conditions, no volumetric work is performed, and the heat released, Qv is equal to the total change in the thermodynamic internal energy of the system, as expressed in Equation (5):

(5) $$Q_{v}=\xi \times {∆}_{r}U_{m}\left(T_{avg}\right)+n_{mix}\times C_{v,mix}\times \left(T_{2}-T_{1}\right)$$

where Δ*_r_U_m_(T_avg_)* is calculated according to Equation (6) (with all quantities expressed in units of J·mol^−1^):

(6) $${∆}_{r}U_{m}\left(T_{avg}\right)={∆}_{r}H_{m}\left(T_{avg}\right)+8.314\times T_{avg}$$

(2) Constant-Temperature Constant-Volume Process

Under this condition, the system temperature remains essentially constant; consequently, the physical enthalpy change and the physical internal energy change are zero. The total enthalpy change (Equation (7)) and the heat released (Equation (8)) are determined solely by the chemical reaction:

(7) $$\Delta H=\xi \times {∆}_{r}H_{m}\left(T_{1}\right)$$

(8) $$Q_{v}=\xi \times {∆}_{r}U_{m}\left(T_{1}\right)$$

where T_1_ denotes the initial reaction temperature, K. Δ*_r_U_m_(T_1_)* is likewise calculated using Equation (6).

#### 2.3.2. Influence of Temperature on Enthalpy Change

**Figure 6:**
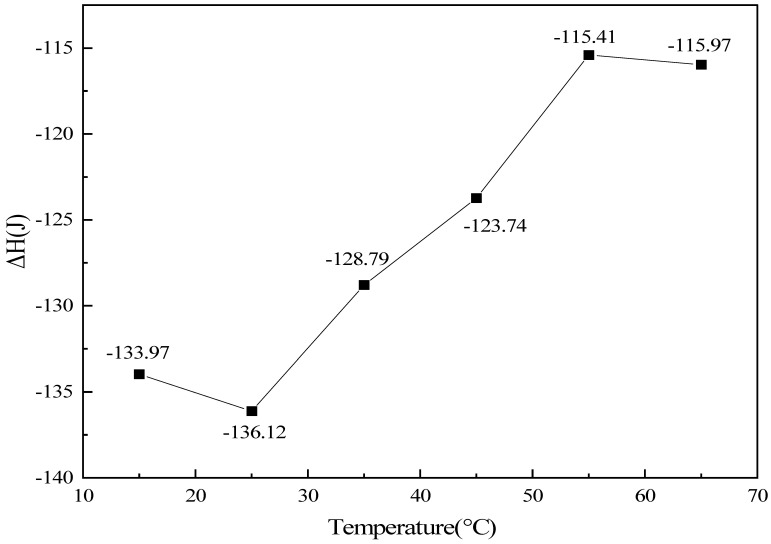
Variation of total enthalpy change with temperature under different experimental conditions (lines are guides for the eye).

Based on the variations in temperature, gas concentrations, and pressure monitored throughout the reaction process, the total enthalpy change during the CO oxidation reaction at different temperatures was calculated according to Equation (4). The results are presented in Figure 6.

Figure 6 illustrates the influence of initial reaction temperature on the total enthalpy change of the CO catalytic oxidation system. Across the entire experimental temperature range of 15–65 °C, the total enthalpy change remained negative, consistent with the intrinsically exothermic nature of the CO oxidation reaction. The data exhibited a three-stage variation characterized by an initial increase, followed by a decrease, and finally a slight recovery, with the maximum absolute value of the total enthalpy change occurring at 25 °C.

In the low-temperature region from 15 to 25 °C, the elevated temperature enhanced the catalytic activity, enabling the reaction to attain the maximum extent of reaction observed in this study. The negative contribution from the intrinsic chemical reaction enthalpy dominated, while the modest temperature rise within the system resulted in a weak offsetting effect from the physical enthalpy change. Consequently, the absolute value of the total enthalpy change reached its peak, underscoring the pronounced promotional effect of temperature on CO oxidation conversion at lower temperatures.

In the intermediate-to-high temperature region from 25 to 55 °C, as the initial temperature increased, the initial absolute amount of CO in the system decreased, leading to a progressive reduction in the extent of reaction. The total contribution of the intrinsic enthalpy change accordingly diminishes. Concurrently, the larger temperature rise amplifies the counteracting influence of the physical enthalpy change, resulting in a continuous decline in the absolute value of the total enthalpy change. This trend reflects a shift in the dominant reaction mechanism from kinetic control toward constraints imposed by material availability and boundary conditions.

Within the range of 55–65 °C, the temperature rise of the system was minimal, and the offsetting effect of the physical enthalpy change became negligible, giving rise to a slight rebound in the absolute value of the total enthalpy change. The consistently negative enthalpy change observed across the full temperature range confirms the thermodynamic feasibility of employing this catalyst for CO oxidation over a wide temperature window. Furthermore, the observed enthalpy variation pattern clearly elucidates the dual regulatory role of temperature—both promotional and constraining—on the CO oxidation reaction.

#### 2.3.3. Influence of Temperature on Heat Release

**Figure 7:**
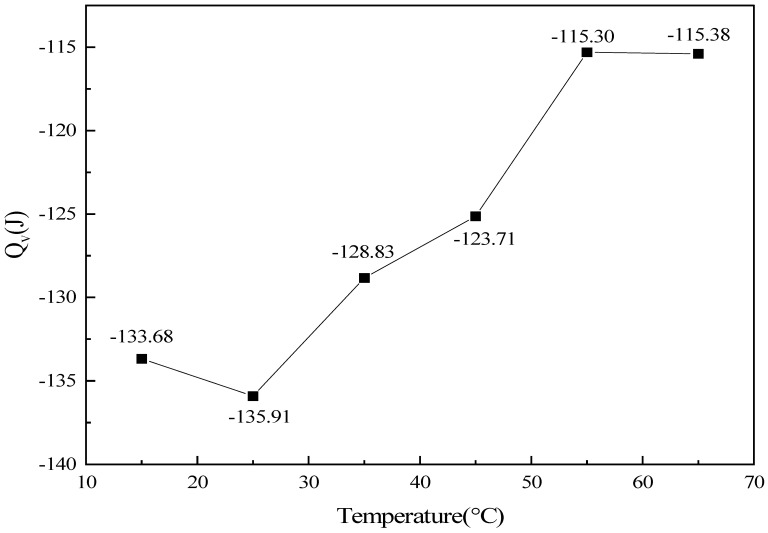
Variation of total heat release with temperature under different experimental conditions (lines are guides for the eye).

Figure 7 presents the influence of the initial reaction temperature on the total heat release of the CO catalytic oxidation system. Under the constant-volume experimental conditions employed herein, the total heat release exhibited excellent numerical agreement and an entirely consistent trend with the total enthalpy change discussed in the preceding section. This close correspondence arises because the pressure fluctuation within the experimental system is minimal, and the contribution of the *PV* term associated with the variation in the total amount of gaseous species before and after the reaction is negligible. Consequently, the total enthalpy change and the constant-volume heat release are nearly equal in magnitude, and both quantities remain negative, thereby directly quantifying the exothermic extent of the reaction.

Across the entire temperature range investigated, the total heat release displayed a three-stage evolution characterized by a slight increase, followed by a sustained decrease, and finally a minor recovery, with the peak exothermic value of −135.91 J occurring at 25 °C. In the low-temperature region from 15 to 25 °C, the increase in temperature enhanced the catalytic activity, enabling the maximum CO conversion amount to be attained under the experimental conditions, and the heat release correspondingly rose to its peak value. Within the intermediate-to-high temperature region from 25 to 55 °C, as the initial temperature increased, the initial absolute quantity of CO within the system declined, leading to a progressive reduction in the extent of reaction and a concomitant decrease in the total heat release. In the range of 55–65 °C, the temperature rise of the system was negligible, and the heat release remained essentially constant, exhibiting only a marginal recovery. The consistently stable exothermic characteristics observed across the entire temperature range confirm the robust heat release performance of this catalyst for CO oxidation over a wide operating temperature window.

### 2.4. Influence of Temperature on Entropy Change (*Δ*S)

Entropy is a fundamental state function that characterizes the degree of microscopic disorder within a system. The molar reaction entropy change of the CO catalytic oxidation reaction governs the temperature dependence of the spontaneous tendency of the reaction, while the total entropy change of the system serves as a key quantitative indicator for evaluating the thermodynamic irreversibility and the variation in disorder during the CO elimination process. Consistent with the above, the total entropy values were used for the confined constant-volume system.

#### 2.4.1. Calculation Method

(1) Constant-Volume Variable-Temperature Process

The molar reaction entropy change is corrected for temperature according to Equation (9):

(9) $${∆}_{r}S_{m}\left(T_{avg}\right)={∆}_{r}S_{m}\left(298.15K\right)+{∆}_{r}C_{p,m}\times \ln\frac{T_{avg}}{298.15}$$

The total entropy change consists of the chemical reaction entropy change and the physical entropy change, as expressed in Equation (10):

(10) $$\Delta S=\xi \times {∆}_{r}S_{m}\left(T_{avg}\right)+n_{mix}\times C_{v,mix}\times \ln\frac{T_{2}}{T_{1}}$$

where ΔS denotes the total entropy change, J·K^−1^; ξ is the extent of reaction, mol; Δ*_r_S_m_* represents the molar reaction entropy, J·mol^−1^·K^−1^; n_mix_ is the initial total amount of substance of the gas mixture, mol; and *C_v,mix_* is the constant-volume heat capacity of the gas mixture, J·mol^−1^·K^−1^.

(2) Constant-Temperature Constant-Volume Process

Under this condition, the system temperature remains essentially constant; hence, the physical entropy change is negligible. The total entropy change is determined solely by the chemical reaction entropy change, as expressed in Equation (11):

(11) $$\Delta S=\xi \times {∆}_{r}S_{m}\left(T_{1}\right)$$

#### 2.4.2. Influence of Temperature on Entropy Change

**Figure 8:**
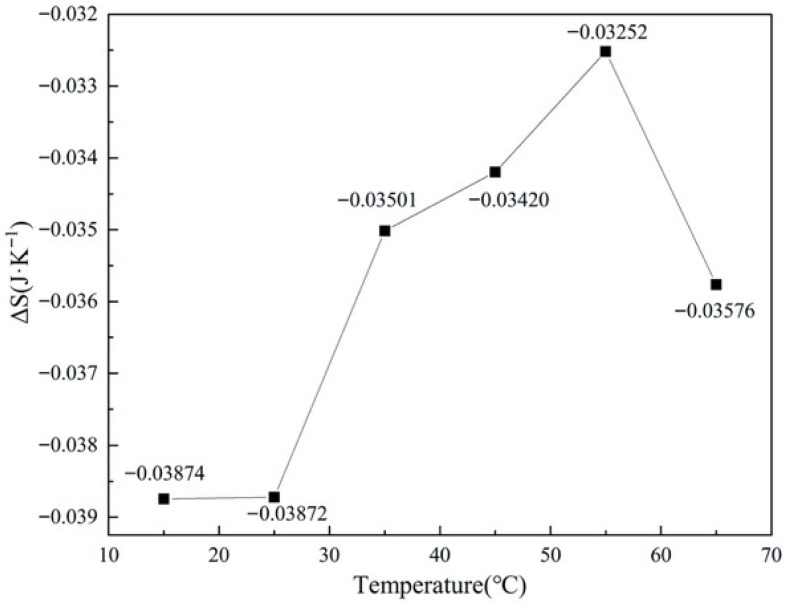
Variation of total entropy change with temperature under different experimental conditions (lines are guides for the eye).

Figure 8 illustrates the influence of the initial reaction temperature on the total entropy change of the CO catalytic oxidation system. Across the entire experimental temperature range of 15–65 °C, the total entropy change remained negative, consistent with the intrinsic thermodynamic characteristics of the CO oxidation reaction—namely, a reduction in the total number of gas molecules and a concomitant decrease in the degree of microscopic disorder within the system. The data exhibited a three-stage variation characterized by an essentially constant level, followed by a continuous decrease, and finally a pronounced recovery.

In the low-temperature region from 15 to 25 °C, the total entropy change remained virtually unchanged, with its absolute value attaining the highest level observed across the entire temperature range. Within this interval, the elevated temperature enhanced the catalytic activity, enabling the extent of reaction to reach its maximum experimental value. The negative contribution from the chemical reaction entropy was absolutely dominant, while the modest temperature rise of the system rendered the positive offsetting effect of the physical entropy change—induced by the temperature increase—extremely weak. Consequently, the absolute value of the total entropy change was maintained at a high level, which is fully consistent with the high degree of CO conversion characteristic of the low-temperature regime.

In the intermediate-to-high temperature region from 25 to 55 °C, as the initial temperature increases, the initial absolute quantity of CO within the system decreases, leading to a progressive reduction in the extent of reaction. The overall negative contribution of the chemical reaction entropy correspondingly diminishes. Simultaneously, the larger temperature rise of the system amplifies the positive offsetting effect of the physical entropy change. These two factors jointly contribute to a continuous decrease in the absolute value of the total entropy change, reflecting a shift in the dominant reaction mechanism from kinetic control toward constraints imposed by material availability and boundary conditions.

Within the range of 55–65 °C, the temperature rise of the system is negligible, and the offsetting contribution of the physical entropy change essentially vanishes. The total entropy change is thus determined solely by the chemical reaction entropy change, resulting in a marked recovery of its absolute value.

The variation pattern of the total entropy change across the entire temperature range exhibits a strong correspondence with the trends observed for enthalpy change and heat release discussed in the preceding sections. This consistency further corroborates the dual regulatory role—both promotional and constraining—of temperature on the CO oxidation reaction process. Moreover, the persistently negative entropy change reaffirms the thermodynamic spontaneity of this reaction over the wide temperature range of 15–65 °C.

### 2.5. Influence of Temperature on the Equilibrium Constant (K)

The standard equilibrium constant, *K*_θ_, is a fundamental parameter that characterizes the thermodynamic conversion limit of a chemical reaction. It depends solely on the reaction temperature and the intrinsic enthalpy and entropy properties of the reaction, and it provides a quantitative description of the theoretical maximum extent of conversion for the CO catalytic oxidation reaction at a given temperature. As such, it constitutes a critical basis for evaluating the limiting extent to which the reaction may proceed spontaneously.

#### 2.5.1. Calculation Method

The molar Gibbs free energy change serves as the essential link between the intrinsic enthalpy and entropy characteristics of the reaction and the equilibrium constant, as expressed in Equation (12):

(12) $${∆}_{r}G_{m}\left(T\right)={∆}_{r}H_{m}\left(T\right)-T\times {∆}_{r}S_{m}\left(T\right)$$

The relationship between the standard equilibrium constant and the molar Gibbs free energy change is given by Equations (13) and (14):

(13) $$\ln K=-\frac{{∆}_{r}G_{m}\left(T\right)}{R\times T}$$

(14) $$K=e^{-\frac{{∆}_{r}G_{m}\left(T\right)}{R\times T}}$$

#### 2.5.2. Influence of Temperature on the Equilibrium Constant

**Figure 9:**
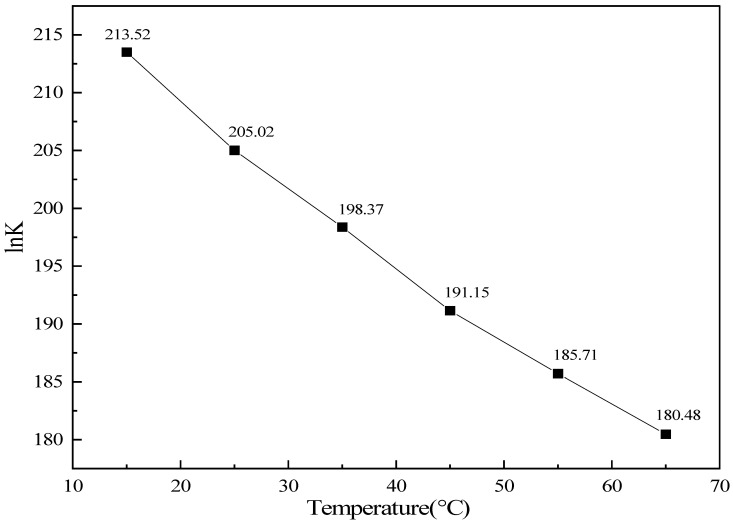
Variation of ln K with temperature under different experimental conditions (lines are guides for the eye).

Figure 9 illustrates the influence of the initial reaction temperature on the natural logarithm of the standard equilibrium constant, ln K, for the CO catalytic oxidation reaction. Across the entire experimental temperature range of 15–65 °C, ln *K* remained positive and resided within an extremely high numerical range, exhibiting a pronounced linear decreasing trend with increasing temperature. This behavior is in full agreement with the intrinsic thermodynamic characteristics of the strongly exothermic CO oxidation reaction. According to the van ’t Hoff isobaric equation, the equilibrium constant of a strongly exothermic reaction decreases as the temperature rises. In the present case, the absolute value of the molar reaction enthalpy change exceeded 560 kJ·mol^−1^, indicating an exceptionally strong exothermic effect. Consequently, an increase in temperature exerts a substantial driving force toward shifting the equilibrium in the reverse direction, resulting in a significant decline in ln *K* with rising temperature.

Although the equilibrium constant decreased considerably as the temperature increased, its magnitude remained exceptionally high throughout the entire temperature range. This can be attributed to two primary factors. First, the molar Gibbs free energy change, ΔrGm, remained a large negative value across the full temperature interval; even at 65 °C, ln K reached 180.48, corresponding to a standard equilibrium constant *K* on the order of 10^78^, which far exceeds the thermodynamic threshold of K > 10^5^ commonly regarded as indicative of a reaction proceeding to completion. Second, the favorable contribution of the strongly exothermic enthalpy change overwhelmingly compensates for the unfavorable entropic effect arising from the reduction in the total number of gas molecules, thereby preserving an extremely strong thermodynamic spontaneity over the entire temperature range. An increase in temperature merely leads to a slight attenuation of the ultimate conversion extent, without altering the essential characteristic of nearly complete conversion.

The consistently ultra-high equilibrium constants observed across the entire temperature range are in excellent agreement with the experimentally measured complete CO conversion, thereby validating the thermodynamic feasibility of employing this catalyst for CO oxidation over the broad temperature interval of 15–65 °C. These findings provide crucial theoretical support for the engineering application of this catalytic elimination technology.

## 3. Experimental Methods

### 3.1. Catalyst Selection

Hopcalite was employed as the catalyst for carbon monoxide oxidation in this study. Hopcalite is a granular catalyst composed of active manganese dioxide (MnO_2_) and copper oxide (CuO) in a mass ratio of approximately 3:2.

### 3.2. Catalyst Characterization

The crystal structure, micromorphology and surface adsorption properties of the hopcalite catalyst were characterized by X-ray diffraction (XRD, Rigaku Ultima IV, Rigaku Corporation, Tokyo, Japan), scanning electron microscopy (SEM, ZEISS MERLIN Compac, Carl Zeiss AG, Oberkochen, Germany), and Fourier transform infrared spectroscopy (FTIR, Tensor37, Bruker, Ettlingen, Germany). XRD analysis was performed over a 2θ range of 10–90° to identify crystalline phases. SEM was used to observe the surface morphology and particle structure of the catalyst. FTIR measurements were carried out to analyze the surface active sites of the catalyst after CO adsorption.

### 3.3. Experimental System

**Figure 10:**
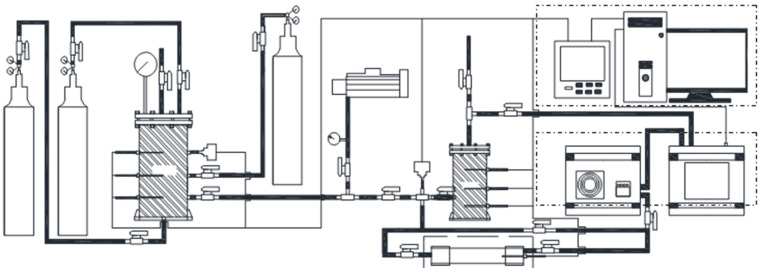
Schematic diagram of the experimental system.

A self-constructed experimental platform was utilized for the CO catalytic oxidation experiments. The experimental system comprised four main sections: (1) Gas supply system: includes gas cylinders and pressure gauges; (2) Reaction system: consists of the elimination vessel and the reaction chamber; (3) Temperature and pressure acquisition system: includes temperature sensors, pressure sensors, and a paperless recorder; (4) Gas analysis system: composed of a condenser and a gas analyzer; and (5) Exhaust system: includes a vacuum pump and exhaust piping. Following the completion of each experiment, the tail gas is vented to the atmosphere, after which the vessel is evacuated to minimize residual carbon monoxide. A schematic diagram of the experimental system is presented in Figure 10.

### 3.4. Experimental Design and Procedure

High-purity CO (99.999%), O_2_ (99.999%), and N_2_ (99.99%) were used to prepare a gas mixture of specified concentration and pressure within a gas-mixing cylinder. After the elimination agent was placed inside the elimination vessel, the vessel was evacuated, and subsequently filled with the gas mixture to an absolute pressure of 0.1013 MPa for the experimental runs. During the experiments, a gas analyzer was employed to monitor and record the real-time variations in CO concentration, CO_2_ concentration, and O_2_ concentration.

The study aimed to investigate the influence of temperature on key thermodynamic parameters during the CO elimination process, including enthalpy change (ΔH), entropy change (ΔS), Gibbs free energy change (ΔG), and the chemical equilibrium constant (K). Furthermore, the underlying thermodynamic processes were analyzed. The experimental conditions are summarized in Table 2.

To minimize experimental errors arising from variations in gas mixture concentration, the total pressure within the gas-mixing cylinder was set to 0.5000 MPa, thereby ensuring that the concentration of the gas mixture remained absolutely identical for each experiment conducted at a given nominal concentration. The experimental system was evacuated to a gauge pressure of −0.0994 MPa, which was considered to represent a vacuum state for the purposes of this study. According to Dalton’s law of partial pressures, the sequential introduction of gases resulted in the following pressure increments: after N_2_ injection, the pressure reached 0.3880 MPa; after O_2_ injection, the pressure attained 0.4850 MPa; and after CO injection, the final total pressure reached 0.5000 MPa.

## 4. Conclusions

Based on the ideal gas assumption and constant-volume reaction boundary conditions, this study systematically evaluated the thermodynamic parameters of hopcalite-catalyzed CO oxidation across the temperature range of 15–65 °C. The findings elucidate, from a theoretical perspective, the regulatory mechanism of temperature on the thermodynamic state functions of a strongly exothermic, entropy-decreasing reaction within a confined system. It should be noted that this confined constant-volume system is fundamentally different from conventional open continuous flow reactors, and the conclusions drawn are only applicable to closed post-disaster emergency scenarios without mass exchange. Overall, the thermodynamic behavior across the entire temperature range confirms that under rigid constant-volume conditions—where external mass exchange and pressure-volume work are precluded—the evolution of thermodynamic parameters strictly adheres to the theoretical expectations of Kirchhoff’s law and the van ’t Hoff equation, yet exhibits distinct non-linear stage-dependent characteristics.

Specifically, from a theoretical standpoint, the occurrence of an extremum inflection point in both the total enthalpy change and total heat release at 25 °C reveals that for a strongly exothermic reaction with an exceptionally large absolute molar reaction enthalpy, the contribution of temperature to the thermodynamic benefit of the reaction transitions from positive promotion to negative constraint when the kinetic gains derived from the temperature rise are insufficient to offset the decline in the extent of reaction caused by the limited absolute quantity of reactants within the constant-volume system. In the analysis of entropy change, although the negative molar reaction entropy exhibits only a weak increasing trend with rising temperature, the absolute value of the total entropy change in the intermediate-to-high temperature region undergoes a sustained decline. This confirms that when evaluating changes in the degree of disorder within a closed system, the offsetting effect of the physical entropy change induced by the temperature rise on the entropy decrease associated with the chemical reaction cannot be disregarded. Furthermore, although the natural logarithm of the standard equilibrium constant exhibits a strictly linear decrease with increasing temperature, its numerical value remains consistently within an extremely high range. For the CO catalytic oxidation reaction, whose intrinsic molar Gibbs free energy change is substantially negative, the equilibrium limit remains far from constituting the theoretical ceiling of the conversion extent even under the thermodynamically least favorable condition of 65 °C. Consequently, any constraint imposed on the ultimate reaction limit by variations in the equilibrium constant can be theoretically excluded.

In summary, through a refined deconstruction of the constant-volume variable-temperature and constant-temperature constant-volume processes, this study not only validates the spontaneous inevitability and exceptionally high conversion limit of the CO elimination reaction within the framework of thermodynamic theory, but more importantly, elucidates how the initial state parameters of the system—under the ideal gas approximation—govern the non-monotonic evolution trajectory of macroscopic thermodynamic parameters by influencing the extent of the reaction and the physical heat capacity compensation term. This work provides a precise computational paradigm and a basis for parameter correction in the thermodynamic analysis of strongly exothermic gas–solid catalytic reactions in confined spaces.

## Figures and Tables

**Table 1:** Fundamental thermodynamic parameters of relevant species at the standard state of 298.15 K and 100 kPa.

Species	Standard Molar Enthalpy of Formation/(kJ·mol^−1^)	Standard Molar Entropy/(J·mol^−1^·K^−1^)	Molar Heat Capacity at Constant Pressure/(J·mol^−1^·K^−1^)
CO (g)	−110.525	197.674	29.14
O_2_ (g)	0	205.138	29.355
CO_2_ (g)	−393.509	213.74	37.11

**Table 2:** Experimental conditions and design parameters.

No.	1	2	3	4	5	6
CO concentration	3%	3%	3%	3%	3%	3%
Temperature	15 °C	25 °C	35 °C	45 °C	55 °C	65 °C

## Data Availability

The raw data supporting the conclusions of this article will be made available by the authors on request.
